# The effects of high-protein vs conventional protein enteral nutrition on intestinal permeability, severity of disease, and mortality in critically ill intensive care unit Patients: Protocol for a randomized controlled trial

**DOI:** 10.1016/j.conctc.2026.101621

**Published:** 2026-02-21

**Authors:** Shonaz Ahmadikhatir, Pardis Irandoost, Omid Moradi Moghaddam, Mohammad Safarian

**Affiliations:** a Department of Nutrition, Faculty of Medicine, Mashhad University of Medical Sciences, Mashhad, Iran; b Department of Nutrition, School of Public Health, Iran University of Medical Sciences, Tehran, Iran; c Nutritional Sciences Research Center, Iran University of Medical Sciences, Tehran, Iran; d Trauma and Injury Research Center, Iran University of Medical Sciences, Tehran, Iran; e Department of Critical Care, School of Medicine, Iran University of Medical Sciences, Tehran, Iran; f Metabolic Syndrome Research Center, Mashhad University of Medical Sciences, Mashhad, Iran

**Keywords:** Intestinal permeability, Intensive care units, Enteral nutrition, Protein, Zonulin

## Abstract

**Background:**

Intestinal permeability is an important determinant in intensive care unit patients. Enteral nutrition is vital for nutritional supplementation in critically ill ICU patients. The amount of Enteral nutrition protein and its impact on intestinal barrier permeability and function are challenging issues. Zonulin is a protein that regulates intestinal epithelial cell tight junction permeability and has the potential to be used as a biomarker for assessing the integrity of the intestinal barrier. However, there are few data from studies on intestinal permeability and the effects of enteral feeding of protein on zonulin levels in critically ill patients. This study was conducted to investigate the effect of high-protein enteral nutrition on intestinal permeability in ICU patients.

**Methods:**

Participants were selected from 88 adult patients with critical illness aged 18-65 years who were admitted to the ICU of Hazrat Rasoul Akram Hospital in Tehran. In this randomized controlled clinical trial, patients will be allocated randomly into two groups of 44. The intervention group will receive high-protein (HP) enteral nutrition (1.6 g/kg/day), and the control group will receive conventional-protein (CP) enteral nutrition (1.2 g/kg/day) for 10 days. The primary outcome will be the change in serum Zonulin levels during the intervention period. Zonulin will be measured on days 0, 5, and 10. The SOFA, APACHE II, and mNUTRIC scores will also be assessed. Mortality will be calculated at 30 and 60 days after the intervention. The secondary outcomes will be SOFA score and 30- and 60-day mortality.

**Discussion:**

Our study aims to present new evidence about the role of protein in enteral nutrition on intestinal permeability in critically ill patients in the ICU.

**Trial registrations:**

The clinical trial was registered on December 11, 2024 in the Iranian Registry of Clinical Trials (IRCT) (IRCT20241129063891N1).

## 1 Background

**Fig. 1:**
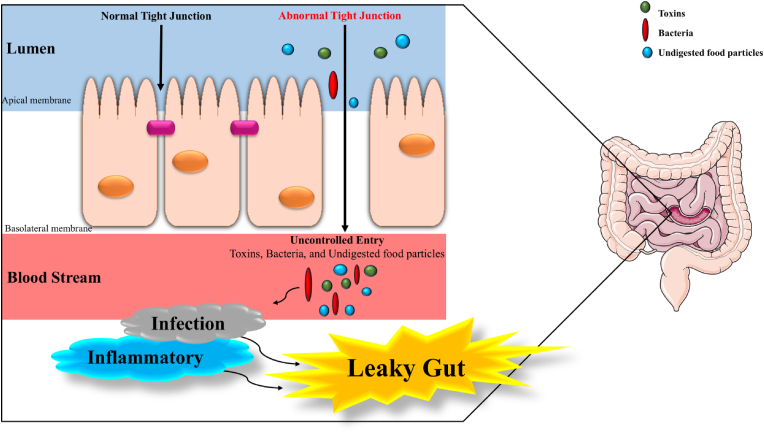
Leaky gut.

Intestinal permeability is the ability of molecules such as bacteria, toxins, or partially digested food particles to pass through the intestinal epithelium and enter the bloodstream [1]. The intestinal barrier normally includes epithelial cells connected by tight junctions. This setup allows selective nutrient absorption and keeps harmful agents out [2]. However, in serious conditions such as sepsis, severe burns, or major trauma, this barrier can become disrupted. This leads to increased intestinal permeability, often called “leaky gut” [3] (see Fig. 1).

Zonulin is a membrane protein made by intestinal epithelial cells. It affects the permeability of tight junctions, which in turn impacts intestinal barrier function [4,5]. Various triggers, such as certain dietary components such as gluten, harmful bacteria, and inflammatory signals, can cause its release. Increased zonulin secretion leads to the destruction of tight junctions, resulting in increased paracellular transport [6]. When zonulin is excessively elevated or chronically elevated, it may contribute to sustained barrier dysfunction and pathological intestinal permeability [7].

The integrity of the intestinal barrier is vital for patients in the intensive care unit (ICU), as its disruption can lead to severe complications [8]. Increased intestinal permeability in these patients facilitates bacterial translocation and endotoxemia, which are related to systemic inflammatory response syndrome (SIRS), sepsis, and multiple organ dysfunction syndrome (MODS) [9]. These complications significantly contribute to increased hospital stay and mortality rates [9,10]. Enteral nutrition (EN), especially protein intake in ICU admission patients, can reduce associated risks and support patient recovery [11].

EN not only serves as a source of essential macro- and micronutrients but also contributes to the preservation of gut barrier function, the modulation of the immune response, and the reduction of inflammation [12]. The protein content and various amino acids in enteral nutrition for ICU patients have been shown to suppress excessive zonulin release, thereby stabilizing tight junctions and reducing intestinal permeability [13]. One study revealed that intestinal permeability damage improved with protein intake during severe heat stress [14]. The consumption of whey protein and glutamine significantly improved intestinal permeability and intestinal morphology in patients with Crohn's disease [15]. Furthermore, the protein component of enteral formulas plays a critical role in intestinal health [16]. In addition to supporting metabolic demands and tissue repair, dietary proteins promote epithelial cell regeneration, tight junction maintenance, mucosal immune defense, and protection against oxidative stress [17]. In ICU patients, who are highly susceptible to gut barrier breakdown and microbial translocation, adequate protein provision is of particular importance for improving clinical outcomes [18]. According to the ESPEN guidelines [19], critically ill patients must be supplied with approximately 1.3 g/kg/day protein. Similarly, the ASPEN guidelines [20] recommend 1.2–2.0 g/kg/day based on disease severity, metabolic stress, and nutritional status. In cases such as severe burns or major trauma, the requirements may increase to 2.5 g/kg/day.

Essential nutrients are vital for maintaining intestinal barrier function. A lack of protein can lead to villous atrophy, reduced expression of tight junction proteins, increased bacterial movement, and increased intestinal permeability. Some studies have shown a significant relationship between intestinal permeability, nutritional support, and protein intake in certain patients. Zonulin, a physiologic controller of intercellular tight junctions, is crucial for protecting against microorganisms in the intestine and is involved in intestinal immunity. An elevated level of zonulin has been reported in septic ICU patients [21]. We hypothesize that protein intake might reduce intestinal permeability and lower circulating zonulin levels in critically ill patients. Studies in critically ill patients are limited. Consequently, our study aimed to investigate the effects of high-protein (HP) versus conventional-protein (CP) intake in enteral nutrition on intestinal permeability, disease severity, and mortality in critically ill patients in the ICU.

## 2 Methods/design

### 2.1 Trial design

**Fig. 2:**
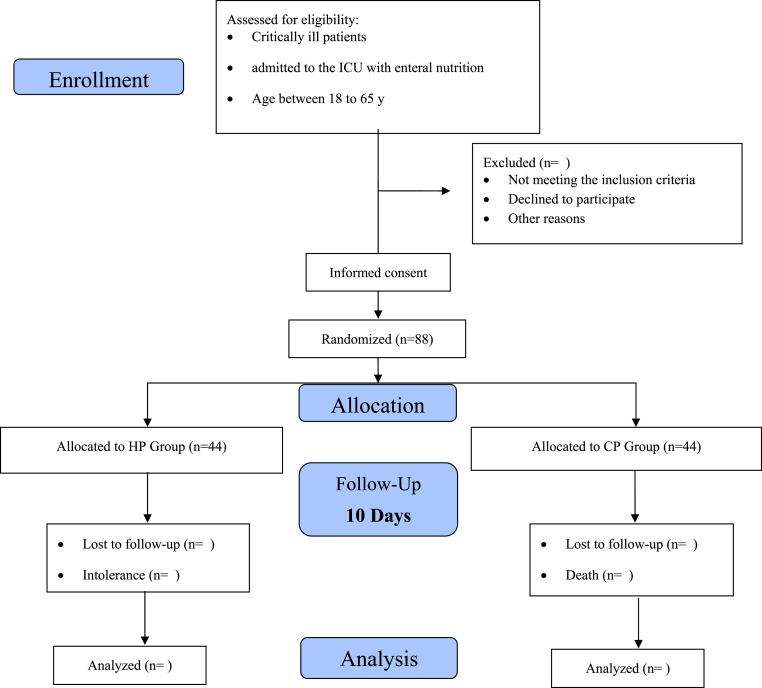
The flowchart of the study protocol.

This study is a randomized, double-blind clinical trial with two groups that receive different doses of protein via enteral nutrition: the CP group and the HP group. All patients will received enteral nutrition through tube feeding. Critically ill patients admitted to the ICU will be eligible for enrollment 24 h after admission. Protein administration begins 48 to 72 h after admission, depending on the patients' suitability. The acute physiology and chronic health evaluation II (APACHE II) and modified nutritional risk in critically ill (mNUTRIC) scores will be assessed at ICU admission. Sequential Organ Failure Assessment (SOFA) score will be evaluated at the beginning and end of the intervention period. Blood samples will be collected on days 0, 5, and 10 for the measurement of zonulin levels and intestinal permeability. Serum zonulin has been widely used in clinical studies as a practical and minimally invasive biomarker reflecting intestinal barrier function [22]. Established tests for intestinal permeability, such as the lactulose/mannitol ratio, are limited in critically ill ICU patients due to practical and clinical constraints, including the need for enteral substrate administration, timed urine collection, and potential hemodynamic instability [23]. Therefore, we used serum Zonulin as a reliable biomarker for intestinal barrier function in ICU patients. [13,21,22,24]. The medication administered during the intervention will be documented and organized by drug class. During the 10-day intervention, patients will be under continuous clinical observation and full monitoring by the treatment team and will be constantly monitored for protein feeding tolerance. The blood samples will be centrifuged and frozen at −70 °C until laboratory analysis. Finally, data on zonulin levels, intestinal permeability, and clinical scores will be analyzed via SPSS and appropriate statistical methods. The trial procedure is illustrated in the flow chart presented in Fig. 2. The protocol was created following the SPIRIT guidelines (see Table 1).

**Table 1:**
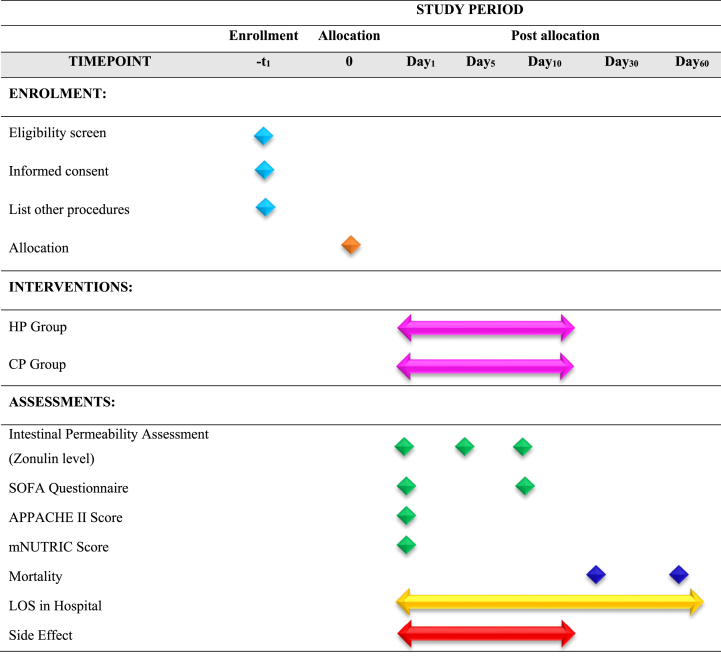
The SPIRIT includes a checklist of enrollment, interventions, and assessments.

### 2.2 Study setting

The clinical trial will be conducted at the ICU of Hazrat Rasool Akram Hospital in Tehran under the joint academic supervision of Mashhad University of Medical Sciences and Iran University of Medical Sciences. Both institutions collaborate in the oversight of study implementation, adherence to ethical standards, and data quality assurance. The study subjects will be critically ill patients admitted to the ICU 24 h after admission. The participants will be selected via a simple random sampling method. Eligible patients will be between 18 and 65 years old and categorized under general ICU admission.

A total of 88 critically ill patients who fulfill all the inclusion and exclusion criteria will be recruited on the basis of previous studies and sample size calculations. The principal investigator (SH. A) After providing informed consent from the patient's legal guardian—following a full explanation of the study's objectives and procedures, emphasizing confidentiality and voluntary participation—patients will be randomly allocated to either the control or intervention group, with 44 patients in each. Recruitment procedures will involve in-person visits to the ICU by the research team. Throughout the study period, all participants will be monitored under the supervision of a board-certified intensive care specialist (O.M.M.).

### 2.3 Eligibility criteria

Individuals will be screened for eligibility, and informed consent will be obtained.

### 2.4 Inclusion criteria

The criteria listed below are required for participation and enrollment in the study.

• Age between 18 and 65 years (inclusive), of either sex
• ICU admission for at least 24 h
• Initiation of EN for a minimum of 24 h prior to enrollment
• Hemodynamically stable at the time of inclusion
• Receiving ≥60% of the prescribed protein requirements via EN (for various reasons, such as severe catabolism, adequate protein intake cannot be achieved via EN alone after 3-7 days, and approximately 60% of the total protein requirement is provided enterally, it is advisable to initiate supplemental parenteral nutrition (SPN) alongside enteral nutrition to provide protein and calorie intake to patients).
• Provision of written informed consent by the patient's legal representative following full disclosure of the study objectives and procedures.

### 2.5 Exclusion criteria

Participants will be excluded from the study if any of the following conditions are met.

• Diagnosis of sepsis at the time of screening
• Presence of chronic conditions such as liver cirrhosis, stage 3 or 4 chronic kidney disease, or acute renal failure (ARF)
• Diagnosis of malignancy with receipt of chemotherapy within the past month
• Known gastrointestinal disorders, including celiac disease, Crohn's disease, ulcerative colitis, or short bowel syndrome
• Use of immunosuppressive agents (e.g., corticosteroids) within one week prior to enrollment
• Use of probiotic supplements within one month prior to enrollment
• Pregnancy or lactation
• Body mass index (BMI) <18 or >40 kg/m^2^

### 2.6 Reasons for eliminating the data

• Expected ICU stay of less than 48 h
• Patient death or discharge before day 4 of the study
• Use of glutamine supplement during the study period
• NPO (nil per os) status for more than 4 consecutive days
• Presence of any absolute contraindication to EN
• Refusal or withdrawal of consent at any stage of the intervention

### 2.7 Randomization and allocation

Given the importance of randomization on the one hand and the need for balanced sample sizes between the studied groups on the other hand, the random permutation block method is used. For this purpose, random permutation blocks of length 4 with two interventions are generated, and patients are allocated to the intervention arms accordingly. Allocation will be concealed in sealed envelopes prepared by an independent researcher.

### 2.8 Blinding

Patients will be assigned to either an HP diet or a CP intake plan. We acknowledge that complete blinding in nutritional intervention trials may be challenging, particularly when individualized protein targets or supplemental parenteral nutrition are required. The nutritional formulations provided in both groups will be visually indistinguishable, share similar color, texture, and packaging, and will be delivered in identical containers labeled with unique codes. Protein dosing will be adjusted by an ICU-based clinical dietician unaffiliated with the research team to ensure healthcare personnel remain blinded. As a result, healthcare personnel, including medical and nursing staff, will be blinded to the nutritional content administered to each participant. Given patients’ critical illness and reduced consciousness, they are not aware of their treatment assignment, which inherently reduces the risk of patient-level bias. If supplemental parenteral nutrition is required, it will be documented and considered in the analysis to minimize bias. Outcome assessments, including serum zonulin measurements, clinical scoring systems such as SOFA scores, and mortality rates, will be assessed by blinded independent raters without knowledge of the groups to which the patients were allocated. APACHE II and mNUTRIC scores were assessed only at ICU admission. All the data will be coded and deidentified prior to statistical processing, and the biostatistician will remain blinded until the final analysis is completed.

### 2.9 Sample size

The sample size was determined on the basis of serum zonulin levels, which were considered the primary outcome of the study. In the study by Klaus et al. [21], the mean zonulin level in the target patient population was reported to be 3.40 ng/mL, with a standard deviation of 0.71. A type I error (α) of 0.05 and a statistical power (1−β) of 80% were used for the calculation. A 10% change in zonulin levels was deemed clinically significant. This threshold was chosen based on previous reports of variation in zonulin levels in critical illness and feasibility considerations for sample size estimation [21]. The exploratory nature of this outcome is acknowledged. Using the standard formula for sample size estimation based on a two-sided independent samples *t*-test to determine the mean of the intervention group versus the control group, the required sample size was calculated to be 41 participants per group, or a total of 82 participants. The final sample size was adjusted to 44 participants in each group, out of a total of 88 participants, considering an expected attrition rate of 5% during the study period.

### 2.10 Objective

#### 2.10.1 Primary objective

The main objective of this study was to investigate the effects of HP versus CP in enteral nutrition on intestinal permeability in critically ill ICU patients.

#### 2.10.2 Secondary objective

The secondary objective was to investigate the effects of HP versus CP in enteral nutrition on illness severity and mortality in critically ill ICU patients.

#### 2.10.3 Intervention

The intervention group will receive HP enteral nutrition at a dosage of 1.6 g/kg/day, whereas the control group will receive CP enteral nutrition at a dosage of 1.2 g/kg/day. The intervention will last for 10 days in ICU-admitted patients.

#### 2.10.4 Primary outcomes

• Zonulin levels will be measured to assess intestinal permeability.

#### 2.10.5 Secondary outcomes

• SOFA questionnaire
• 30-day and 60-day mortality rates
• Length of stay (LOS) in a hospital

#### 2.10.6 Confounding variables

Potential confounding variables include age, sex, BMI, APACHE II score at ICU admission, admission diagnosis category, vasopressor use during the intervention, and underlying comorbidities. Comorbidities will be categorized into predefined clinical groups, including Trauma, cancer, surgery, diabetes mellitus, kidney disease, cardiovascular disease, and liver disease. These factors should be measured and considered, as they may influence outcomes.

#### 2.10.7 Protein in enteral

The amount of protein administered is based on the patient's body weight. A portion of the protein requirement is met through conventional enteral nutrition formulas, while the remaining portion is supplemented with whey protein. To prevent an increase in osmolarity and enhance patient tolerance, less than 10 g of protein are administered in divided doses throughout the day.

#### 2.10.8 Enteral protein administration

Gavages will be administered by personnel blinded to the baseline assessments. Once each patient's hemodynamic status is stabilized, tube feeding (via a nasogastric tube or gastrostomy) is initiated. High-protein feeding begins 48 h after ICU admission and continues until either ICU discharge or day 10 of the study. Enteral feeding will be given intermittently every 3 h. The feeding volume will be determined on the basis of each patient's caloric requirements and tolerance. During the first 24 h, 30% of the total caloric need will be initiated. If well tolerated, the volume was gradually increased to the target level. The protein and energy goals should be increased gradually and achieved within the first 48 to 72 h to avoid overfeeding.

#### 2.10.9 Energy requirements

Sufficient energy is necessary to maintain vital functions, prevent muscle catabolism, and support patient recovery. Energy restriction during the initial days is necessary to reduce the risk of refeeding syndrome. We should carefully monitor energy intake, especially in patients at risk of malnutrition. For mechanically ventilated patients, excessive energy intake may lead to increased CO_2_ production, impaired ventilation, and prolonged dependence on the ventilator. In addition to energy, adequate attention must be given to protein requirements, which are critical for preserving lean body mass, as well as fluid and electrolyte balance. During the first 3 to 5 days of hospitalization, energy intake should begin at 15–20 kcal kg/day. Once hemodynamic stability is achieved, intake should be gradually increased to 25–30 kcal/kg/day [19]. Additionally, energy requirements should be adjusted on the basis of individuals’ BMI, as illustrated in the table below.

**Table:** 

BMI (kg/m^2^)		Body weight	
<18.5			Initial: 15–20 kcal/kg Target: 25–30 kcal/kg
18.5- 29.9		Actual Body Weight	25-30 kcal/kg
>30	30-50	Actual Body Weight	11-14 kcal/kg
	>50	Adjusted Body Weight	22-25 kcal/kg

#### 2.10.10 Demographic questionnaire

A demographic questionnaire will also be completed, including age, sex, ethnicity, education level, marital status, occupation, smoking status, special diets, comorbidities, medications, and the use of dietary supplements. Trained personnel will take anthropometric measurements, including weight, height (or ulna length), and arm circumference.

#### 2.10.11 Zonulin

Zonulin levels will be measured in blood samples. Blood samples (5 ml) will be taken from patients before the intervention and again on days 5 and 10 to measure zonulin levels. After centrifugation, the serum will be separated. Zonulin levels in the serum sample will be measured via a sandwich ELISA. The method involves incubating a sample with a specific antibody against zonulin, followed by subsequent detection of the bound zonulin using a secondary antibody and a colorimetric substrate.

#### 2.10.12 Apache II score

The APACHE II score is used to classify patients according to disease severity and assess their baseline health status before admission to the ICU and includes the acute physiology score (which is based on 12 physiological variables), age adjustment, and chronic health evaluation. The total score ranges from 0 to 71. Higher scores can be associated with greater disease severity and risk of mortality.

#### 2.10.13 SOFA score

The SOFA score assesses six major organ systems**,** such as cardiovascular, nervous, respiratory, coagulation, renal, and hepatic systems. In each system, a score of 0 indicates normal functioning, a score of 4 indicates organ dysfunction, and the total score ranges from 0 to 24. Higher SOFA scores are associated with a poorer prognosis, indicating greater degrees of organ dysfunction, poorer clinical outcomes, and increased mortality risk**.**

#### 2.10.14 mNUTRIC score

The NUTRIC questionnaire is the first nutritional risk assessment tool specifically validated for patients in the ICU. This study used a modified version of the NUTRIC score (excluding interleukin-6). The mNUTRIC score ranges from 0 to 9 and is calculated on the basis of clinical data collected during the first 24 h after ICU admission. Scores of 4 or higher indicate high nutritional risk, whereas scores below 4 indicate low nutritional risk.

#### 2.10.15 Mortality

Mortality will be followed up on days 30 and 60 postintervention, with the use of the hospital information system (HIS) by entering the patient's national ID or through phone contact.

#### 2.10.16 Statistical analysis

The data will be analyzed via SPSS version 25. The normality of the data will be assessed with the Kolmogorov‒Smirnov test. Descriptive statistics will include the mean ± SD for normally distributed variables and the interquartile range (IQR) for nonnormally distributed variables. Pre- and postintervention measures will be compared using a paired *t*-test, whereas frequency tables and medians will describe categorical and continuous data. Categorical variables will be analyzed via the chi-square test or Fisher's exact test, and continuous variables will be analyzed via the Mann‒Whitney *U* test. Survival analysis (Kaplan‒Meier and Cox regression) will be used for time‒to‒event outcomes, and ANCOVA will compare primary outcomes at baseline, day 5, and day 10. Potential confounders including age, sex, BMI, APACHE II score at ICU admission, admission diagnosis category, vasopressor use during the intervention, and predefined comorbidity groups will be included as covariates in multivariable regression and ANCOVA models. These adjusted analyses will be applied to both primary (zonulin levels) and secondary outcomes where appropriate to minimize residual confounding. Longitudinal regression models will assess the intervention's effect, controlling for the same confounding factors. Statistical significance will be set at p < 0.05.

## 3 Discussion

This study is the first clinical trial that examined the effects of high-protein enteral nutrition on intestinal permeability, disease severity, and mortality in critically ill patients in the ICU. Critically ill ICU-admitted patients are often at risk of sepsis, inflammation, and impaired intestinal barrier function [25]. These patients are also prone to malnutrition and systemic inflammation, which lead to poor outcomes and increased mortality rates [26].

Nutritional support, especially enteral nutrition and protein intake, can effectively improve epithelial barrier function and reduce intestinal permeability in these patients [16].

Zonulin is a membrane protein that plays a role in maintaining tight junctions between intestinal epithelial cells (through binding to proteins such as claudin, occludin, junctional adhesion molecules, and the actin cytoskeleton) and is considered a marker of epithelial barrier function [27]. The production of this protein is usually regulated by inflammatory cytokines such as IL-1, IL-6, and TNF-α and is increased during inflammatory conditions [28].

The gastrointestinal microbiome can also influence the clinical status of critically ill patients [29]. Dysbiosis or an imbalance in the microbiome is associated with increased zonulin secretion, impaired intestinal permeability, and inappropriate regulation of inflammatory mediators [30]. Therefore, enteral nutrition and protein intake can significantly affect the disease process, epithelial barrier function, and intestinal permeability [16].

The SOFA score is a suitable tool for predicting survival and ICU discharge [31,32]. The APACHE II score also estimates the severity of the disease on the basis of data from the first 24 h of hospitalization [33]. The mNUTRIC questionnaire is also used as a screening tool for the risk of severe malnutrition in ICU patients [34,35].

The present study aims to identify a scientific gap by investigating the effects of high protein administration in enteral nutrition on serum zonulin concentrations, severity of illness scores (APACHE II, SOFA), nutritional risk, and mortality in ICU patients. If protein administration causes a significant reduction in zonulin levels and improved clinical outcomes, the results of this trial could influence future guidelines on protein dosing in enteral nutrition in ICU patients [13].

These findings support the implementation of personalized nutritional strategies aimed at optimizing not only the energy supply but also gut health and systemic status in critical care settings [36,37]. Future studies could also examine the impact of protein on the gastrointestinal microbiome and inflammatory markers to better elucidate the broader immunoinflammatory role of nutrition in the gut‒brain axis in critically ill patients.

## CRediT authorship contribution statement

**Shonaz Ahmadikhatir:** Writing – review & editing, Writing – original draft, Investigation. **Pardis Irandoost:** Writing – review & editing, Methodology. **Omid Moradi Moghaddam:** Validation, Supervision, Project administration. **Mohammad Safarian:** Validation, Supervision, Project administration, Conceptualization.

## Trial registration

This trial was registered on December 11, 2024 in the Iranian Registry of Clinical Trials (ID: IRCT20241129063891N1). This document represents the first version of the study protocol, finalized in Decmber 2025. Participant recruitment has not yet commenced and is anticipated to begin within the next month. The estimated duration for data collection is approximately 12 months.

## Ethics approval and consent to participate

The study protocol followed the SPIRIT checklist. This trial was approved on 2024-11-17 with the ethics code of IR. MUMS.REC.1403.298 at Mashhad University of Medical Sciences. It is also registered with the Iranian Clinical Trials Registry (IRCT20241129063891N1). This trial will be performed in accordance with the Declaration of Helsinki. We will obtain informed consent from the patient's legal guardian, and the study objectives and procedures will be fully explained, with an emphasis on confidentiality and voluntary participation. The results will be published without mentioning the names or characteristics of the study participants. All patients benefit from their standard routine treatment, and no treatment is removed from the patients' routine care.

## Funding

Financial support for this research was granted by the Vice Chancellor of 10.13039/501100004748Mashhad University of Medical Sciences and the Trauma and Injury Research Center of 10.13039/100012021Iran University of Medical Sciences.

## Declaration of competing interest

The authors declare that they have no known competing financial interests or personal relationships that could have appeared to influence the work reported in this paper.

## Data Availability

No data was used for the research described in the article.
